# Multiple Bioactivities of Peptides from Hydrolyzed *Misgurnus anguillicaudatus*

**DOI:** 10.3390/molecules28062589

**Published:** 2023-03-13

**Authors:** Baojie Dou, Xudong Wu, Zihan Xia, Guanghao Wu, Quanyou Guo, Mingsheng Lyu, Shujun Wang

**Affiliations:** 1 Jiangsu Key Laboratory of Marine Bioresources and Environment/Jiangsu Key Laboratory of Marine Biotechnology, Jiangsu Ocean University, Lianyungang 222005, China; 2 Co-Innovation Center of Jiangsu Marine Bio-Industry Technology, Jiangsu Ocean University, Lianyungang 222005, China; 3 East China Sea Fishery Research Institute, Chinese Academy of Fishery Sciences, Shanghai 200090, China

**Keywords:** *Misgurnus anguillicaudatus* (loach), peptides bioactivity, antioxidants, enzymes inhibitor, molecular docking

## Abstract

*Misgurnus anguillicaudatus* (loach) is a widely distributed benthic fish in Asia. In this study, the alkaline protease was used to hydrolyze loach, and the hydrolysate products of different molecular weights were obtained by membrane separation. In vitro antioxidant assays showed that the <3 kDa fraction (SLH-1) exhibited the strongest antioxidant activity (DPPH, hydroxyl radical and superoxide radical scavenging ability, and reducing power), while SLH-1 was purified by gel filtration chromatography, and peptide sequences were identified by LC-MS/MS. A total of six peptides with antioxidant activity were identified, namely SERDPSNIKWGDAGAQ (D-1), TVDGPSGKLWR (D-2), NDHFVKL (D-3), AFRVPTP (D-4), DAGAGIAL (D-5), and VSVVDLTVR (D-6). In vitro angiotensin-converting enzyme (ACE) inhibition assay and pancreatic cholesterol esterase (CE) inhibition assay, peptide D-4 (IC50 95.07 μg/mL, 0.12 mM) and D-2 inhibited ACE, and peptide D-2 (IC50 3.19 mg/mL, 2.62 mM), D-3, and D-6 acted as pancreatic CE inhibitors. The inhibitory mechanisms of these peptides were investigated by molecular docking. The results showed that the peptides acted by binding to the key amino acids of the catalytic domain of enzymes. These results could provide the basis for the nutritional value and promote the type of healthy products from hydrolyzed loach.

## 1. Introduction

Antioxidant compounds are important for human health as they prevent or reduce excessive free radicals and reactive oxygen species (ROS) produced by the body, thus reducing damage to DNA, proteins, and lipids [1,2,3]. Antioxidants and enzyme inhibitors are employed as part of the control strategy for the management of healthy [4]. Metabolic diseases have always been a problem for modern people [5]. Among them, hypertension and hypercholesterolemia are metabolic disorders of the body, and oxidative stress is thought to be involved in their pathogenesis [2,3,5]. ROS are considered to be mediators of blood pressure caused by an angiotensin-converting enzyme (ACE) [3]. Bioactive peptides are defined as specific protein fragments that have a positive impact on body functions [6]. Bioactive peptides are generally formed from 2–20 amino acid units and perform multiple health benefits such as antioxidant [7], antihypertensive [8], hypoglycemic [9], cholesterol lowering effect [10], immunomodulatory [11], antifatigue [12], and antiaging activities [13]. Bioactive peptides, such as antioxidant peptides, are rich in aromatic amino acids, acidic amino acids, and hydrophobic amino acids that contribute to antioxidant properties [14]. Likewise, these amino acids can facilitate the formation of hydrogen bonds, hydrophobic interactions, and other interactions with ACE and pancreatic cholesterol esterase (CE) to inhibit enzyme activity and exert health-promoting effects [10,15].

A parent protein contains inactive but specific amino acids, and the protein is able to demonstrate its potential biological activity only when these amino acids are released through processes, such as in vitro hydrolysis of peptide bonds [16]. Enzymatic hydrolysis causes some improvement in the functional properties of proteins and has the advantages of being rapid, safe, easily controllable, and low cost [17]. Rona et al. [18] used pepsin-chymotrypsin-trypsin to enzymatically decompose pigeon pea seeds, and hydrolysis peptides showed strong ACE inhibitory activity and antioxidant activity. Priti [19] et al. used papain to hydrolyze camel milk protein to obtain three pancreatic CE inhibitory peptides. Animal and plant proteins are the source of bioactive peptides [8]. Due to the excellent nutritional profile, good amino acid composition, and beneficial biological activity of fish hydrolysates, their potential industrial applications as functional food ingredients have been extensively investigated [20], such as sardine hydrolysates [21] and sturgeon skin hydrolysates [22], which were shown to have antioxidant effects. Furthermore, Khawaja et al. [23] obtained 10 antioxidant peptides from mackerel muscle hydrolysates. The peptide ALSTWTLQLGSTSFSASPM had the highest DPPH scavenging activity, and the peptide LGTLLFIAIP had the highest SOD-like activity. Furthermore, researchers have applied modern bioinformatic techniques, such as molecular docking techniques and molecular dynamics simulations, to explain the molecules mechanism of bioactive peptides [24,25].

*Misgurnus anguillicaudatus* (loach) is widely distributed in a benthic river in Asia [26] and is a common traditional freshwater food fish in China and Korea [27]. Loaches are small commercial fish, and its meat is very delicious [28,29]. According to the China Fishery Statistical Yearbook, loach productions continuously increased at an average annual rate of 5.7% in recent years, and loach production reached 367,428 tons in 2021 [30]. The meat of loach is rich in nutrients and contains many healthful active substances, and the Chinese discovered its medicinal value in ancient [31]. Loach has been studied for the lectins, antimicrobial peptides, polysaccharides, and glycoproteins purified from its mucus [26]. Loach proteins have recently received considerable research attention, and the studies are mainly focused on active peptides of these proteins for their antioxidant, antifatigue, and anticancer cell proliferative properties. You et al. [32] hydrolyzed loach with papain to obtain crude peptides with antifatigue, antioxidant, and anticancer cell proliferative effects. In addition, the new antioxidant sequence Pro–Ser–Tyr–Val was produced from loach [31].

The aim of this study was to isolate antioxidant active peptides from protease hydrolyzed loach with a variety of biological activities that could be beneficial to human health (ACE inhibitory activity and pancreatic CE inhibitory activity). The mechanism of action of the peptides was studied by molecular docking and experiments. Our results will provide a basis for determining the nutritional of loach value of human health. In addition, it will broaden the type of products of processed loach.

## 2. Results and Discussion

### 2.1. Barrier Separation

**Figure 1:**
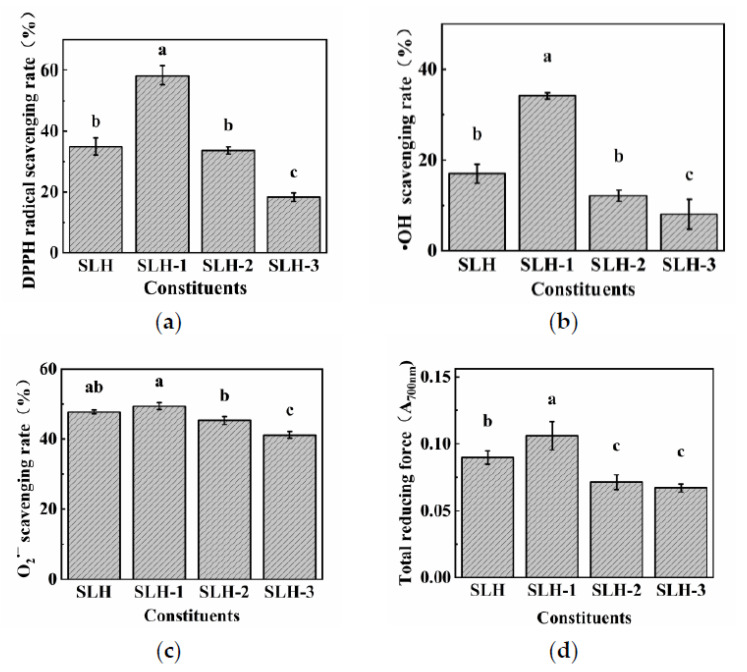
Antioxidant activity of various fraction of hydrolysates of loach (**a**) DPPH radical scavenging rate of various fraction of hydrolysates of loach; (**b**) Hydroxyl radical scavenging rate of various fraction of hydrolysates of loach; (**c**) Superoxide radical scavenging rate of various fraction of hydrolysates of loach; (**d**) Total reducing force of various fraction of hydrolysates of loach. The different letters represent the statistically significant difference (*p* < 0.05).

**Figure 2:**
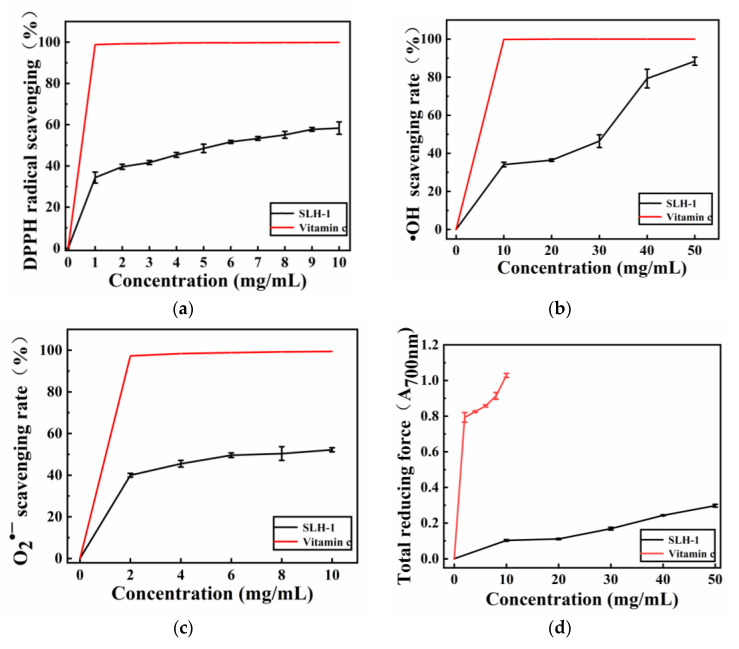
Antioxidant activity of SLH–1 (**a**) DPPH radical scavenging rate of SLH–1; (**b**) Hydroxyl radical scavenging rate of SLH–1; (**c**) Superoxide radical scavenging rate of SLH–1; (**d**) Total reducing force of SLH–1.

As shown in Figure 1, at a concentration of 10 mg/mL, the DPPH, hydroxyl radical, and total reducing power of SLH–1 were 58.34%, 34.15%, and 0.106, respectively, which were significantly higher than those of SLH, SLH-2, and SLH-3. Many studies have shown that the enzymatic products of protein and low-molecular-weight peptides can effectively inhibit the interaction of free radicals [17]. Studies have also shown that the DPPH scavenging activity of SLH–1 at different mass concentrations increased with the concentration (Figure 2a). When the SLH–1 concentration reached 5.53 mg/mL, the DPPH clearance rate reached 50%. DPPH is a relatively stable lipid-free radical, and antioxidants could inhibit the oxidation of free radicals [33]. DPPH inhibition and reduction have been extensively used to assess the antioxidant capacity of substances [34]. As shown in Figure 2b, SLH–1 exhibited dose-dependent hydroxyl radical scavenging activity at 10–50 mg/mL, with an IC50 value of 30.66 mg/mL. Superoxide anion, which is produced through aerobic respiration, is a signaling molecule that is particularly crucial for regulating apoptosis and senescence [35]. The superoxide radical scavenging activity was 39.96% at 2 mg/mL SLH–1 (Figure 2c). The IC50 value was calculated to be 7.46 mg/mL, which indicated that SLH–1 had a good superoxide radical scavenging activity. The total reducing force absorbance of SLH–1 was 0.298 at a concentration of 50 mg/mL (Figure 2d). The absorbance continuously increased with an increase in mass concentration, which indicates a good restoring force. The loach protein was hydrolyzed by protease to form small-molecule polypeptides, which exhibited good antioxidant activity. The biological activity of peptides is affected by their molecular weights. The membrane separation technology can be used to enrich peptides of a certain molecular weight [36]. Different molecular sizes of peptides may contribute to their different biological activities and functional properties [37], and overall, low-molecular-weight peptides have more biological activity [38]. Our results are similar to those of Zhong [39], who extracted bioactive peptides from tuna hydrolysates.

### 2.2. G–25 Gel Chromatographic Separation

**Figure 3:**
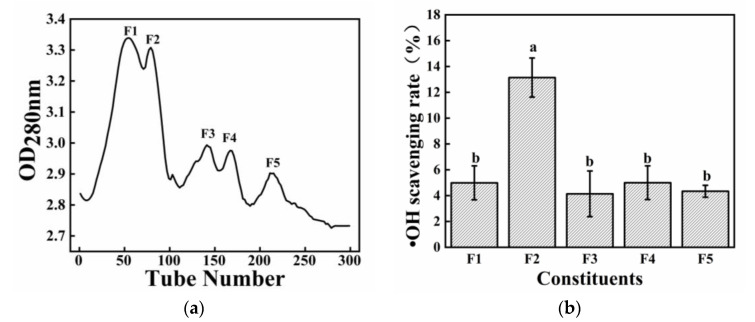
(**a**) Elution curve for SLH–1 in G25; (**b**) Hydroxyl radical scavenging activity of each elution peak. The different letters represent the statistically significant difference (*p* < 0.05).

Figure 3a presents the results of the sample separation of SLH–1 according to the molecular size of the peptide by using a G25 chromatographic column. The peptide was separated into five components, and the hydroxyl radical scavenging activities of these components are presented in Figure 3b. Component F2 (Tube Number: 71–111, second peak) exhibited the strongest hydroxyl radical activity compared with other components. At 0.35 mg/mL, Component F2 showed the strongest hydroxyl radical scavenging activity, indicating that the antioxidant activity is related to the molecular weight. Other studies have purified antioxidant peptides similar to those found in the present study [17]. The relationship between the functional properties and structure of the active peptide was clarified by further identifying the amino acid sequence in Component F2 through LC–MS/MS.

### 2.3. Peptide Identification

We identified the bioactive peptides in F2 through the LC–MS/MS polypeptide sequence analysis, and a total of 535 peptides were identified. The six peptides with the highest score in the total peptides were chosen (Table 1), which were SERDPSNIKWGDAGAQ (D-1), TVDGPSGKLWR (D-2), NDHFVKL (D-3), AFRVPTP (D-4), DAGAGIAL (D-5), and VSVVDLTVR (D-6)) (The spectra of these peptides are in Figures S1–S3). The different bioactive functions of a peptide are related to factors, such as the composition and sequence of amino acids, and the type and size of amino acids at the amino or carboxyl ends [16]. These factors determine the solubility and hydrophobicity of peptides and directly affect their activity and their ability to be absorbed by the body [40]. The secondary structure of the six peptides was predicted to be a random coil by online website. It has been shown that the biological activity of peptides were related to their structure, and the increase in the random coil of their secondary structure enhanced their antioxidant activity [41]. The higher the proportion of irregular curl, the looser the structure of the peptide, which can better expose the active site and thus bind to the receptor [41,42].

### 2.4. Biological Activity of Synthetic Peptides

#### 2.4.1. Antioxidant Activity

**Figure 4:**
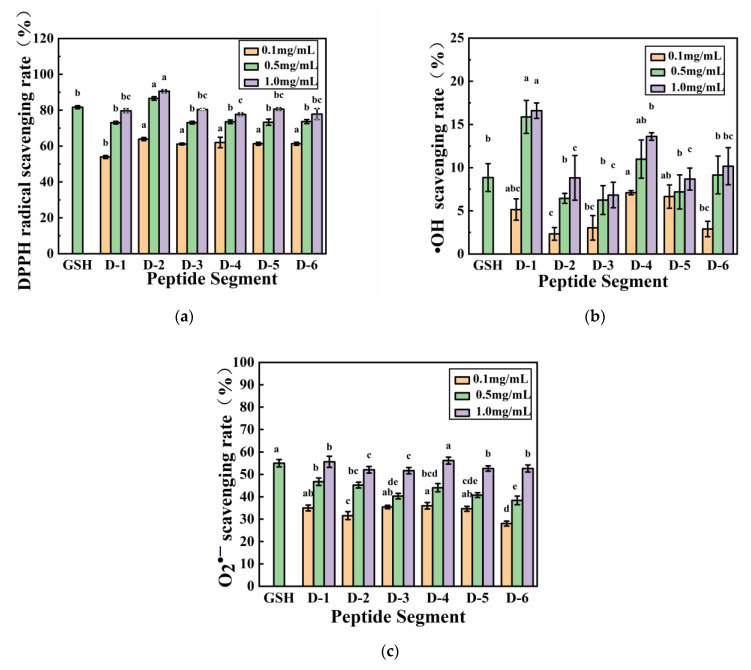
Antioxidant activity of the six peptides (**a**) DPPH clearance rate of the six peptides; (**b**) Hydroxyl radical scavenging rate of the six peptides; (**c**) Superoxide radical scavenging rate of the six peptides. The different letters represent the statistically significant difference (*p* < 0.05).

As shown in Figure 4a, the DPPH scavenging activity of peptide D–2 was 86.57% at 0.5 mg/mL, which was higher than that of glutathione at the same concentration. This indicated that Peptide D–2 had stronger DPPH scavenging activity than glutathione. At 0.5 mg/mL, the DPPH scavenging activity of Peptides D–1, D–3, D–4, D–5, and D–6 reached more than 90% of that of glutathione at the same concentration. Thus, D–1, D–3, D–4, D–5, and D–6 had excellent DPPH scavenging activity similar to glutathione, and with an increase in concentration, the DPPH scavenging activity increased. The peptide extracted from the loach had a high DPPH scavenging activity. Figure 4b shows that the hydroxyl radical scavenging activities of peptides D–1, D–4, and D–6 at 0.5 mg/mL were 15.87%, 10.99%, and 9.15% higher than that of glutathione at the same concentration. As shown in Figure 4c, although the superoxide anion and reducing power of the six peptide segments were weaker than those of glutathione at 0.5 mg/mL, the superoxide anion scavenging activity of these six peptide segments increased with an increase in concentration. At 1 mg/mL, the superoxide radical scavenging capacity of all the six peptides exceeded 50%, exhibiting different antioxidant activities. Studies have shown that the antioxidant activity of peptides is closely related to the hydrophobic amino acids contained in those peptide species [33]. Acidic amino acids have an ability to donate electrons to free radicals and have been proven to strongly contribute to DPPH clearance [14,33]. All the peptides synthesized in the present study, except D–4, contained Asp. In addition, aromatic amino acids are considered the main antioxidant amino acids that are a crucial player in antioxidant activity [43,44].

#### 2.4.2. ACE Inhibitory Activity

**Figure 5:**
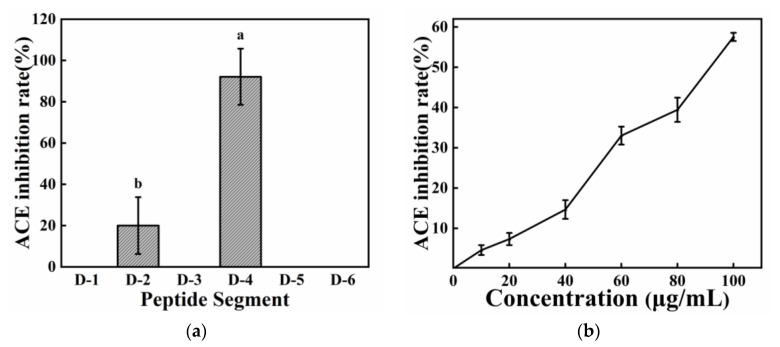
ACE scavenging activity (**a**) ACE scavenging activity of the six peptides; (**b**) ACE scavenging activity of D–4. The different letters represent the statistically significant difference (*p* < 0.05).

Peptide segments D–2 and D–4 exhibited ACE inhibitory activity, and the inhibitory activity of D–4 was significantly higher than that of D–2, reaching 92.12% (Figure 5a). The bioactivity of protein hydrolysis products is dependent on the amino acid composition, sequence, and configuration of peptides [16,45]. The sequence and configuration of peptides are related [46]. The hydrophilic–hydrophobic ratio in the peptide sequence is the key factor for ACE inhibitory activity, and ACE is believed to prefer inhibitors containing hydrophobic amino acid residues at the C-terminal position [45]. Moreover, the C-terminal proline residue most likely enhances the peptide’s inhibitory activity against ACE because the imidazole ring of Pro residues and the electron cloud of the hydrophobic interaction easily bind to the amino acid residues at the ACE active site [47]. The peptide AFRVPTP had the same C-terminal proline residue as lisinopril and enalapril [48], and its IC50 for ACE inhibition was 95.07 μg/mL(0.12 mM) (Figure 5b). Furthermore, D–2 contained the aromatic residue tryptophan in its C-terminal tripeptide sequence. Peptides containing branched aliphatic amino acids such as glycine, valine, leucine, and isoleucine at the N–terminal position have been reported to exhibit ACE inhibitory activity [45,49]. In addition, the C-terminal with Arg greatly promotes the potent ACE inhibitory activity of long-chain peptides, and D–2 contained R at the C-terminal, which improved its ACE inhibitory activity [47]. The hydrophobic amino acid leucine (L) and the positively charged amino acid (K) present in the peptide fragments favored the ACE inhibitory activity of the peptide [46,49].

#### 2.4.3. Pancreatic CE Inhibitory Activity

**Figure 6:**
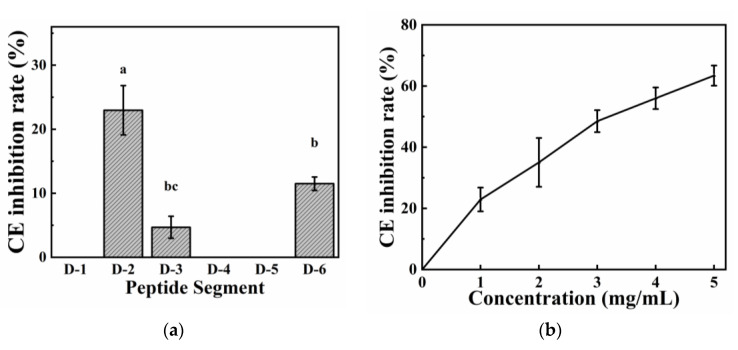
CE scavenging activity (**a**) CE scavenging activity of the six peptides; (**b**) CE scavenging activity of D–2. The different letters represent the statistically significant difference (*p* < 0.05).

CE is a polymerase produced by pancreatic acinar cells. It plays a key role in hydrolyzing cholesterol esters in diet, thus producing cholesterol and free fatty acids [50]. Figure 6a depicts the inhibitory effect of these six peptides on pancreatic CE at a concentration of 1 mg/mL. The inhibitory activity of D–2 was significantly higher than those of D–3 and D–6. D–2 showed more effective inhibition, and its inhibitory effect on CE increased as its concentration increased, with an IC50 value of 3.19 mg/mL (2.62 mM) (Figure 6b). Peptides with pancreatic CE inhibitory activity were obtained and predicted from camel milk and amaranth protein [2,19,51]. To date, no study has investigated the CE inhibitory activity of loach peptides in in vitro studies. This study provides a reference for future researchers regarding the knowledge of loach protein hydrolysates to produce anti-hypercholesterolemic peptides.

### 2.5. Molecular Docking

Nrf2/Keap1 pathway is the key system, regulating expression of antioxidant and cytoprotective genes. The Keap1-Nrf2-ARE-signaling pathway directly regulates various antioxidant enzymes and GSH synthesis-related enzymes as well as detoxification enzymes [52]. The inhibition of the Keap1–Nrf2 interaction would activate the downstream oxidation resistance of this pathway and the expression of genes encoding cytoprotective proteins, thereby improving the body’s antioxidant capacity [33]. Enhancing Nrf2 activity by disrupting the Keap2–Nrf1 interaction is one way to develop therapeutic anti-oxidative stress drugs [53]. Therefore, we predicted the antioxidant potential of the peptide in vivo by molecular docking of the peptide with Keap1.

**Figure 7:**
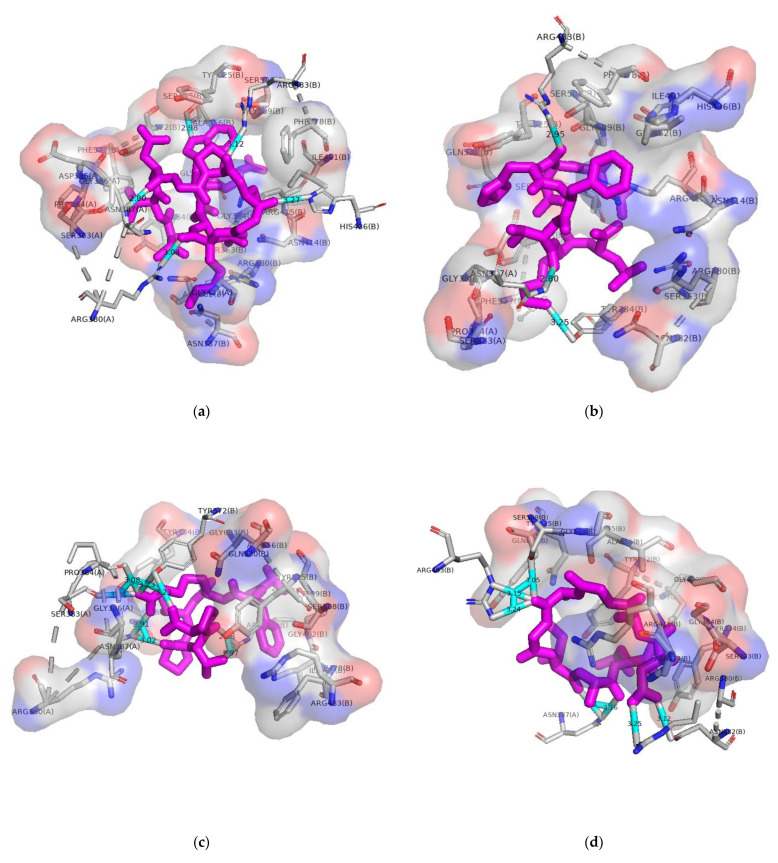

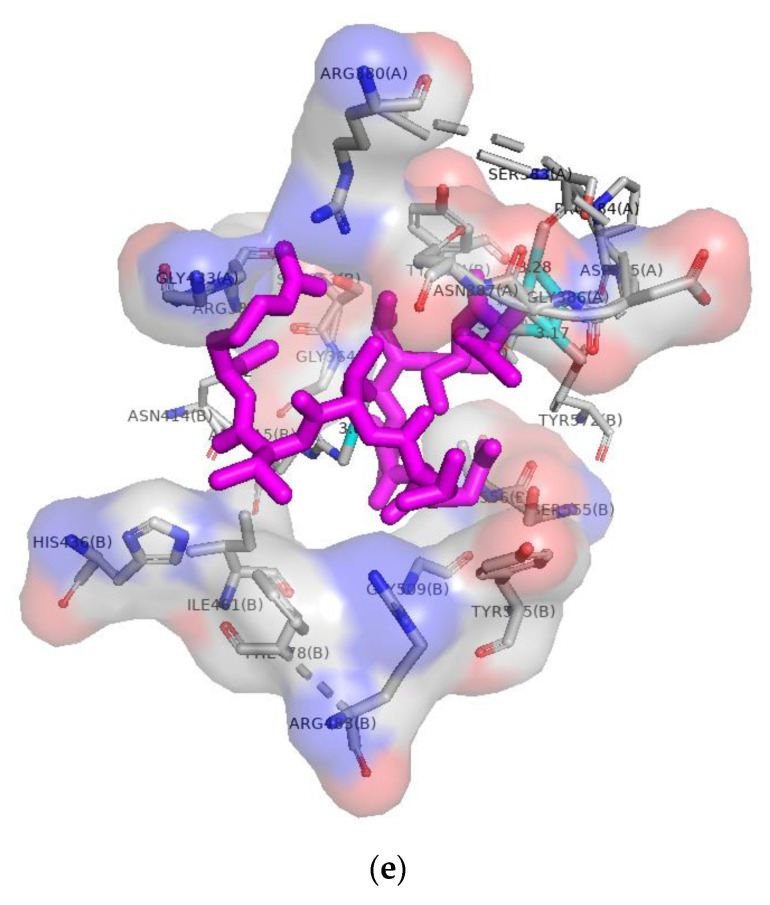
Molecular docking of peptides with Keap1 (**a**) Molecular docking of D–2 to Keap1; (**b**) Molecular docking of D–3 to Keap1; (**c**) Molecular docking of D–4 to Keap1; (**d**) Molecular docking of D–5 to Keap1; (**e**) Molecular docking of D–6 to Keap1. The purple bar structure in the diagram represents the peptide and the cyan bar structure represents the hydrogen bond.

In our present study, the binding ability of the loach peptide to Keap1 was assessed through molecular docking. The six peptides, except for D–1, for which docking failed, were ranked as D–4, D–5, D–6, D–3, and D–2 in terms of docking energy strengths, which were −9.0, −8.0, −7.4, −7.3, and −6.4 kcal/mol (Table 2), respectively. This indicated that the five antioxidant peptides could spontaneously bind to Keap1 protein. As shown in Figure 7, the five loach antioxidant peptides could be embedded in the active cavity of Keap1. All five ligands formed hydrogen bonds and hydrophobic bonds with Keap1, thereby forming a stable docking conformation. D–2 generated a total of five hydrogen bonds, including one bond with Arg483 and Arg380 residues of Keap1 [54], and it formed a hydrophobic bond with Asn387, Tyr572, Arg415, and Asn487 active residues [55,56]. These residues are also reported as key residues for the binding of Keap1 to Nrf2. D–3 also forms three hydrogen bonds with key residues Tyr334, Arg483, and Asn387, which are used to inhibit the binding of Keap1 to Nrf2 [33,43,44,55]. With Phe577, Tyr572, Ser555, Arg380, Arg415, Tyr525, and Gln530 [33,43,44,55], D–4 formed a hydrophobic bond. D–4 formed two hydrogen bonds with each of Asn387, Arg415, and Arg415 residues of Keap1 [33,43,44,55], and a hydrophobic bond with the active residues Tyr334, Arg380, Gln530, Tyr525, Arg483, and Ser508 [33,43,44,55] of Keap1. D–4 produced a total of eight hydrogen bonds, and its docking energy was considerably higher than that of other peptide segments. The ligand interacted with the receptor through different intermolecular forces, such as hydrophobic force, Van der Waals force, hydrogen bond, π bond, and electrostatic interaction, with the hydrogen bond interaction being the strongest. D–5 formed seven hydrogen bonds with Keap1, including two with Arg387 and Arg483, and one with Ser508 [33,43,44,55]. These three residues were also the key for the binding of Keap1 to Nrf2. D–6 formed six hydrogen bonds with Keap1, including with the active residues Ser602 and Arg415 [33,43,44,55] of Keap1, with the bond lengths of 3.00 Å and 3.09 Å, respectively. These results indicated that the five peptide fragments might competitively bind to and release Nrf2 from Keap1. Moreover, they exhibit in vivo antioxidant activity by activating the Keap1-Nrf2-ARE pathway. Many food-derived peptides have the potential to activate and release Nrf2 by competitively binding to Keap1 and inhibiting Keap1-Nrf2 interactions. Han [57] formed stable hydrophobic bonds between key amino acid residues of Keap1 protein and the antioxidant peptides obtained from hydrolysis of tuna roe, which may regulate the Keap1-NrF2 pathway. In the study by Zang [55], the novel antioxidant peptide produced through gastrointestinal digestion in snakehead (*Channa argus*) soup acted as an antioxidant by activating the cellular antioxidant Keap1-Nrf2 signaling pathway.

**Figure 8:**
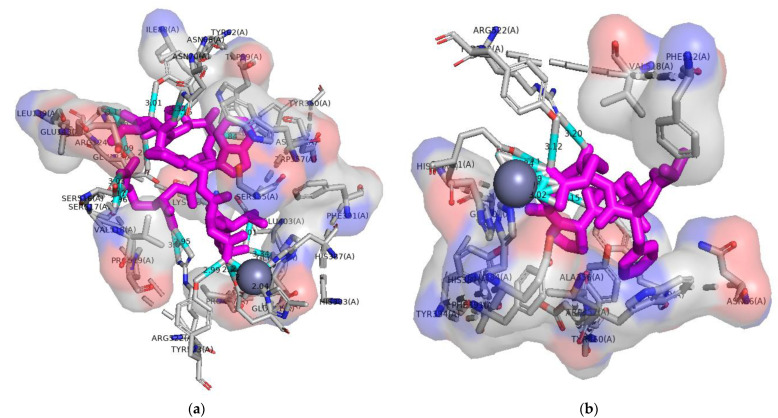
Molecular docking of peptides with ACE (**a**) Molecular docking of D–2 to ACE; (**b**) Molecular docking of D–4 to ACE. The purple bar structure in the diagram represents the peptide and the cyan bar structure represents the hydrogen bond. The gray pellets represent Zn(II).

ACE has three active sites (S1, S2, S1^,^) and Zn(II) [58], which are considered important for its inhibition. Among them, Pocket S1 contains three amino acids (Ala354, Glu384, and Tyr523), S2 contains five amino acids (Gln281, His353, Lys511, His513, and Tyr520), and S1^,^ (Glu162) contains one amino acid; these were considered the main active residues for interaction [45,47,48]. Our results showed that the D–4 peptide had a docking binding energy of −9.1 kcal/mol with ACE (Table 3), and it formed hydrogen bonds with Tyr523, a residue of the S1-binding vesicle with a bond length of 3.12 Å. Moreover, it formed two hydrogen bonds with Glu411, a Zn(II) ligand (Figure 8b). The peptide has a hydrophobic interaction with Glu384 (S1-bound vesicle) and His387, a Zn(II) ligand. In addition, the peptide can directly interact with Zn(II) of ACE. According to the literature, VL–9 and LL–9 can coordinate with Zn(II) of ACE and can twist the tetrahedral geometry, resulting in ACE inhibitory activity [45]. The D–2 peptide had a docking energy of −8.8 kcal/mol and formed a hydrogen bond with Tyr523 in the S1-binding pocket. Similarly, D–2 was observed to interact directly with Zn(II) and the ligand Glu411 of Zn(II) (Figure 8a). Although D-2 produced more hydrogen bonds than D–4, its docking energy was weaker than that of D–4. Furthermore, D–2 had fewer sites of interaction with ACE active residues than D–4, which explained that the ACE inhibitory activity of D–4 was significantly higher than that of D–2. The two peptides, D–2 and D–4, obtained in this study were considered good ACE inhibitors. These inhibitors inhibited the ACE enzyme activity by blocking its active site.

**Figure 9:**
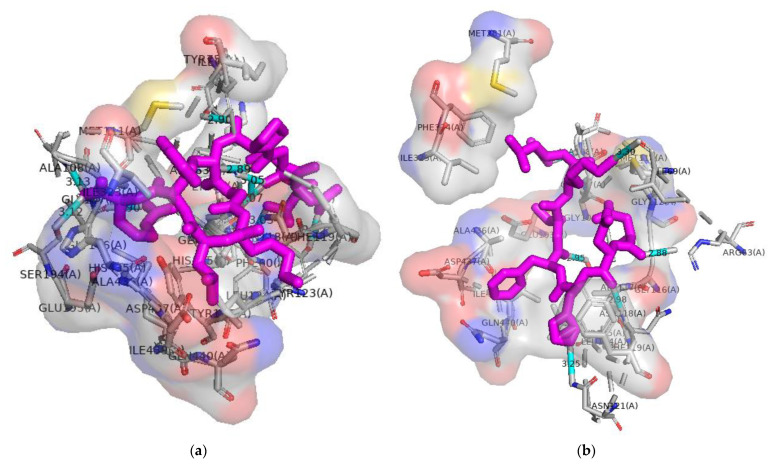

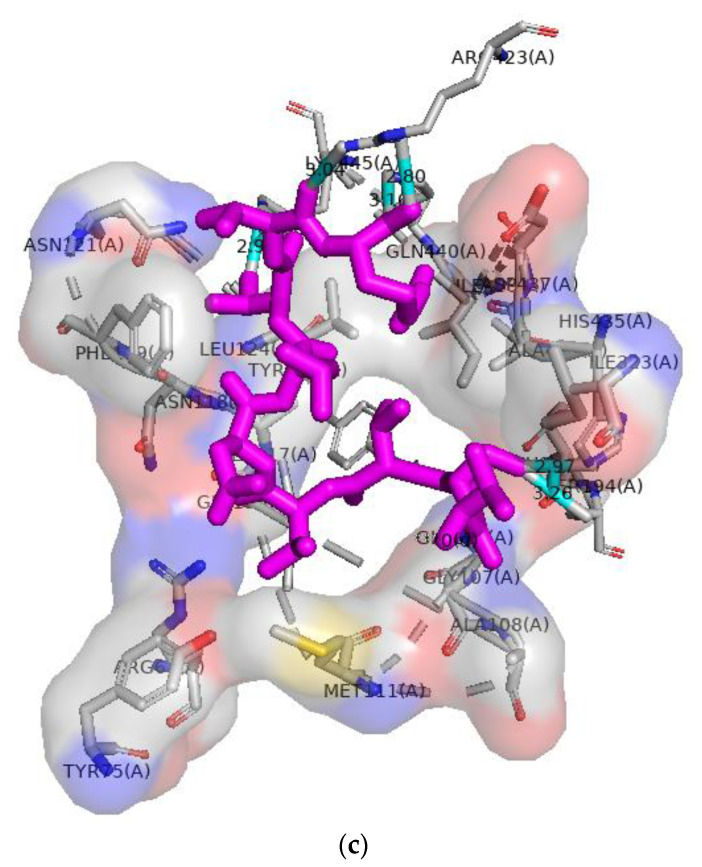
Molecular docking of peptides with CE (**a**) Molecular docking of D–2 to ACE; (**b**) Molecular docking of D–3 to CE; (**c**) Molecular docking of D–6 to CE. The purple bar structure in the diagram represents the peptide and the cyan bar structure represents the hydrogen bond.

CE is a bile salt-activated lipase that hydrolyzes cholesteryl esters, fat-soluble vitamins, triglycerides, and phospholipids [19], and it belongs to the α/β hydrolase folding family. The active site of CE contains catalytic triplets, including Ser194, Asp320, and His435, as well as oxygen anion vacancies (Gly107, Ala108, and Ala195) [19,51], which form tetrahedral intermediates at the active site of the store; these intermediates are essential for its catalytic function. The mechanism of peptide inhibition of pancreatic CE was investigated using an active site-based molecular docking technique. Pancreatic CE from humans was inhibited by peptides D–2, D–3, and D–6 with docking energies of −7.4, −6.9, and −7.0 kcal/mol (Table 4), respectively. As shown in the Figure 9, these three peptides exhibited hydrogen and hydrophobic interactions. According to the docking results, peptides D–2 and D–6 formed hydrogen bonds with one of the catalytic triplet residues. Among them, peptide D-2 formed hydrogen bonds with Ser194, Gly107, and Ala107, and D–6 formed one hydrogen bond with Ser194. In addition, D–3 formed a hydrogen bond with Ala108, as well as a hydrophobic bond with Gly107. The loach protein-hydrolyzing peptide could bind to the active site of pancreatic CE and thus inhibit its activity.

### 2.6. The Safety of Bioactive Peptides

The safety of bioactive peptides is an important basis for their application [59]. Bioactive peptides should be investigated for their allergenicity and toxicity before they were used in functional foods or pharmaceutical formulations [60]. Maeno et al. [61] reported that Val-Pro-Pro, a bioactive tripeptide derived from cow milk, has been shown to have no toxicity to specific target organs. Studies have shown the allergenicity was linked between the molecular weight of peptides and the amino acid composition [62]. The six peptides we obtained from the loach were all non-toxic peptides (Table 1). D–1, D–4, and D–6 were predicted to be non-allergens; however, D–2, D–3, and D–6 were predicted to be possible allergens. Therefore, the further determine their safety by experiments should be carried out before application.

## 3. Materials and Methods

### 3.1. Materials and Chemicals

Loach was purchased from Jinshuiwan Foodstuff Co., Ltd., Lianyungang, China. After washing the loaches with pure water, whole loaches were collected, ground twice in a meat grinder, preserved in polyethylene bags, transported to the laboratory at 4 °C, and subsequently stored in a −20 °C refrigerator until use.

ACE, N-[3-(2-furylacryloyl)]-1-phenylalanyl-glycyl glycine (FAPGG), p-nitrophenyl butyrate (pNPB), and pancreatic CE were purchased from Sigma Co., St. Louis, MO, USA. All other chemicals and reagents are of analytical grade and can be purchased from the market.

### 3.2. Preparation of Loach Protein Hydrolysate

Add 200 g of crushed loach to the reactor. Then, the alkaline protease (Heshibi Biotechnology Co., Yinchuan, China) was added to the reactor at a ratio of 600 U/g of loach meat. To this mixture, deionized water was added to ensure the ratio of loach meat to water was 1:20. The mixture was hydrolyzed at 50 °C and pH 8.0 for 24 h. Then, the hydrolysate was soaked in a bath of boiling water for 15 min to inactivate the enzyme. Finally, the sample was centrifuged at 4 °C and 5000× *g* for 30 min, and the supernatant of the hydrolysate was filtered for later use [31].

### 3.3. Membrane Separation

The supernatant of loach hydrolysate (SLH) was fractionated through membranes with molecular weight cutoffs of 10,000 and 3000 Da, and the intercepted and permeate solutions from each membrane were collected. The solutions of three fractions with different molecular weights, namely SLH-1 (<3000 Da), SLH-2 (3000–10,000 Da), and SLH-3 (>10,000 Da), were obtained and lyophilized before storage at room temperature, and the mass of each lyophilized fraction was weighed.

### 3.4. Antioxidant Activity

#### 3.4.1. DPPH Scavenging Activity

The sample (0.2 mL) was added to 0.2 mL of 0.1 mM of DPPH solution in absolute ethanol and then placed in the dark. OD_517nm_ (Thermo Fisher, Waltham, MA, USA) of the mixture was measured after 30 min [63]. DPPH scavenging activity was calculated as follows:

(1) $$\mathrm{DPPH}\;\mathrm{clearance}=\left(1-\frac{A_{x}-A_{x0}}{A_{0}}\right)\times 100$$

where A_x_ is the absorbance of DPPH reacting with the peptide, A_x0_ is the absorbance of the reaction between ethanol and the peptide, and A_0_ is the absorbance of DPPH and water.

#### 3.4.2. Hydroxyl Radical Reduction

The sample (0.1 mL) was mixed with 0.1 mL of 9 mM FeSO_4_, 0.1 mL of 9 mM of salicylic acid in absolute ethanol, and 0.1 mL of 0.03% H_2_O_2_ and placed in a water bath at 37 °C for 15 min. The absorbance value was determined at 510 nm. Ultrapure water was used as a blank control [64]. The extent of hydroxyl radical reduction was calculated as follows:

(2) $$\mathrm{Hydroxyl}\;\mathrm{radical}\;\mathrm{clearance}=\left(1-\frac{A_{x}-A_{x0}}{A_{0}}\right)\times 100$$

where A_x_ is the absorbance of the sample, A_x0_ is the absorbance of the blank reagent, and A_0_ is the absorbance of the control without the sample.

#### 3.4.3. Superoxide Anion Scavenging Activity

The sample (0.2 mL) was mixed with 1 mL of Tris–HCl (50 mM, pH 8.2) and incubated at 25 °C for 10 min. Then, 30 μL of pyrogallol (6 mM) was immediately added to the mixture and left at room temperature for 30 min, and OD_320nm_ of the mixture was measured. Ultrapure water was used as a blank control [65]. Superoxide anion scavenging activity was measured as follows:

(3) $$\mathrm{Superoxide}\;\mathrm{anion}\;\mathrm{clearance}=\left(1-\frac{A_{x}-A_{x0}}{A_{0}}\right)\times 100$$

where A_x_ is absorbance of the sample, A_x0_ is the absorbance of the sample without pyrogallol, and A_0_ is the absorbance of the blank control.

#### 3.4.4. Determination of Reducing Power

The sample (0.1 mL) was mixed with 0.1 mL of 0.2 M PBS solution (pH 6.6) and 0.1 mL of 1% (*w*/*v*) K_3_Fe(CN)_6_. This mixture was incubated at 50 °C for 20 min. Then, 0.1 mL of 10% trichloroacetic acid was added to the mixture. The mixture was centrifuged at 5000× *g* for 10 min, and 0.05 mL of the supernatant obtained was mixed with 0.25 mL of distilled water and 0.01 mL of 0.2% FeCl_3_. The mixture was then incubated at room temperature for 10 min, and the absorption was measured at 700 nm [65].

### 3.5. G25 Chromatographic Purification

To purify the peptide, the Sephadex G–25 molecular exclusion column (100 cm × 1.6 cm, Xiamei Shanghai, China) was used for fractionation. The void volume of the column was 15 mL. SLH-1 (50 mg /mL) was filtered through a 0.22-filter membrane and loaded into the column. Deionized water was used for balance. The elution flow rate was 1 mL/min, and 300 components were collected. The absorbance of each component was measured at 280 nm, and the highest absorbance peaks were selected and combined. At 0.35 mg/mL, each selected peak was measured to evaluate the hydroxyl radical scavenging activity [39].

### 3.6. Peptide Identification

Peptide sequences were identified for the highest antioxidant activity component purified by G25 chromatographic. The Li [34] approach is slightly modified. The samples were first subjected to reductive alkylation, and then, the processed samples were analyzed through liquid chromatography–tandem mass spectrometry (LC–MS/MS) to obtain raw files of the raw mass spectrometry results. The capillary high-performance Liquid Chromatograph model is Easy–nLC 1200 (Thermo Fisher, Waltham, MA, USA). The capillary liquid chromatography condition was loaded sample volume: 5 μL; mobile phase: A: 0.1% formic acid in water, B: 20% 0.1% formic acid in water—80% acetonitrile; total flow rate: 60 nL/min; LC linear gradient: from 6% to 9% B for 5 min, from 9% to 14% B for 15 min, from 14% to 30% B for 30 min, from 30% to 40% B for 8 min, and from 40% to 95%. The Q Exactive™ hybrid quadrupole–Orbitrap™ Mass Spectrometer (Thermo Fisher, Waltham, MA, USA) had a spray voltage of 2.2 kV and a capillary temperature of 270 °C. These raw results were analyzed using the software Byonic, which searched databases for existing peptides and matched them to the results obtained. The secondary structure of these peptides was predicted by online sites SOPMA (https://prabi.ibcp.fr/htm/site/web/app.php/aboutUs/links (accessed on 4 March 2023)).

### 3.7. Peptide Synthesis

DG peptides Co., Ltd. (Wuhan, China) synthesized the identified peptides using the solid-phase method [34]. The sequence of synthesis was from the C–terminal to the N–terminal of the peptide chain. The synthesized peptides were purified through HPLC. The purity and quality of the purified peptides were determined through LC–MS/MS. The purity of each synthesized peptide was confirmed to be >98%.

### 3.8. ACE Inhibitory Activity

The ACE inhibitory activity was determined according to the experimental method of Hu et al. [66] with slight modification. The sample (100 μL), ACE (50 μL, 0.1 U/mL, prepared from 80 mM borate buffer pH 8.3), and FAPGG (50 μL, 1 mM) in borate buffer HEPES (80 mM, pH 8.3) were added to each well of a 96-well plate. The blank group was replaced with 100 μL of borate-buffered HEPES. The absorbance values of blank and sample groups a_1_ and b_1_ were initially read at 340 nm (Thermo Fisher, Waltham, MA, USA). The sample groups were then reacted at 37 °C for 30 min. Subsequently, the absorbance values of blank and sample groups a_2_ and b_2_ were again read at 340 nm.

(4) $$\mathrm{ACE}\;\mathrm{inhibition}\;\mathrm{rate}\;\left(\%\right)=\frac{A-B}{B}\times 100$$

where A = a_1_ − a_2_, B = b_1_ − b_2_.

### 3.9. Pancreatic CE Inhibitory Activity

The pancreatic CE inhibitory activity was determined according to the experimental method of Mudgil et al. [51] with slight modification. Each sample (50 μL) was incubated at 37 °C with pNPB (50 μL, 5mM) and pancreatic CE (50 μL, 5 μg/mL) in sodium phosphate buffer (0.1 M, pH = 7.2) for 30 min. In the control group, samples were replaced by PBS. For the control blank, samples and pancreatic lipase were replaced by PBS. For the sample blank group, pancreatic CE was replaced by PBS. All absorbance measurements were read at 405 nm by using a microplate reader (Thermo Fisher, USA).

(5) $$\mathrm{CE}\;\mathrm{inhibition}\;\mathrm{rate}\;\left(\%\right)=1-\frac{C-D}{A-B}\times 100$$

where A represents control, B represents control blank, C represents sample, and D represents sample blank.

### 3.10. Molecular Docking

According to a previous method, the X-ray crystal structure (PDB code: 4l7B) of human Keap1 was obtained from the RCSB protein database (PDB) (https://www.rcsb.org/ (accessed on 10 October 2022 )) through molecular docking prediction of the peptide and Keap1. The water molecules and original ligands in the receptor molecule were removed using PyMol 2.5 software and then saved as PDB files. Then, the PyMOL-treated receptor molecule was opened for hydrogenation by using Autodock Tools 1.5.6 software (Scripps Research, USA) and saved as a PDBQT file for later use. The 2D structure of the selected peptide was drawn using ChemDraw 20.0, while the 3D structure was drawn using Chem3D 20.0. These structures were optimized for energy minimization. The 3D structure output was a file in mol2 format. These peptides were defined as ligands. Autodock Vina 1.1.2 was used for docking (center grid box: x = −2.636, y = 3.267, z = −27.433; 40 × 40 × 40 grid points and 0.375× spacing). Unless otherwise specified, default values were adopted for all other parameters. Finally, Ligplot Plus 2.2.5 and PyMol 2.5 were used to visually analyze the molecular docking results [43]. Autodock Vina 1.1.2 was also used to dock ACE (PDB code: 1086; center grid box: x = 43.82, y = 38.31, z = 46.65; 66 × 66 × 66 grid points and 0.375× spacing) [67]. It is worth noting that Zn^2+^ should be retained when the ACE is treated before docking. The docking parameters of Pandian et al. were used for molecular docking of peptide with pancreatic CE (PDB code: 1F6W; center grid box: x = 1.581, y = 8.301, z: = 12.885; 126 × 126 × 124 grid points and 0.375× spacing) [68].

### 3.11. Study on the Safety of Peptides

The safety of peptides is a key factor that restricts their utilization by humans [69]. The potential toxicity (https://webs.iiitd.edu.in/raghava/toxinpred/design.php/ (accessed on 10 October 2022))and sensitization (http://www.ddg-pharmfac.net/AllergenFP/ (accessed on 10 October 2022)).of each synthetic peptide were predicted using online tools. Support vector machine (SVM) and the default SVM threshold of 0.0 were chosen for ToxinPred toxicity prediction. Peptides with an SVM score < 0.0 were predicted as non-toxic [59].

### 3.12. Statistical Analysis

Each experiment was repeated at least three times, and data were determined using one-way analysis of variance (ANOVA) by SPSS (SPSS Statistics version 26, International Business Machine, Armonk, NY, USA) software. Different letters indicated significant differences among different groups (*p* < 0.05).

## 4. Conclusions

In this study, the most potent antioxidant active ingredient F2 was isolated from the loach hydrolysate through ultrafiltration and G25 chromatographic purification, and its peptide profile was characterized through LC–MS/MS. Six novel antioxidant peptides were obtained. All the six peptides displayed good antioxidant activity. More importantly, D–4 (IC50 95.07 μg/mL,0.12 mM) and D–2 exhibited inhibitory effects on ACE, and D–2 (IC50 3.19 mg/mL, 2.62 mM), D–3, and D–6 exhibited inhibitory effects on pancreatic CE. The molecular docking study revealed that peptides may exert its antioxidant activity by occupying the active pocket of keap1 protein, and it may inhibit ACE and CE by binding to the active site. As a natural antioxidant with ACE and CE inhibitory effects, the loach peptide has a broad application potential in functional food products because of its nutritional and health effects.

## Figures and Tables

**Table 1:** Information on six peptides.

Number	Peptide Sequences	Peptide Length	Score	Scan Time	Intensity	pI	Grand Average of Hydropathy	Toxicity	Anaphylaxis
D-1	SERDPSNIKWGDAGAQ	16	557.90	22.14	64,628,000	4.56	−1.42	No	No
D-2	TVDGPSGKLWR	11	524.20	23.70	78,281,000	8.41	−0.79	No	Possible
D-3	NDHFVKL	7	456.80	22.93	193,440,000	6.74	−0.47	No	Possible
D-4	AFRVPTP	7	444.60	31.85	4,081,200	9.79	0.06	No	No
D-5	DAGAGIAL	8	437.10	35.57	49,091,000	3.80	1.18	No	Possible
D-6	VSVVDLTVR	9	412.70	35.28	6,303,100	5.81	1.23	No	No

**Table 2:** Peptide docking with Keap1.

Number	Docking Energy	Number of Hydrogen Bonds	Hydrogen Bonds	Hydrophobic Interactions
D-2	−6.4 kcal/mol	5	Asn387 (A), Ser555, Arg380 (A), His436 (B), Arg483 (B)	Asn382 (B), Tyr334 (B), Pro384 (A), Ser383 (A), Gly386 (A), Phe577 (B), Gly433 (A), Asn387 (A), Arg380 (B), Asn414 (B), Phe478 (B), Ser508 (B), Ile461 (B), Tyr525 (B), Gly509 (B), Gly603 (B), Gly364 (B), Ala556 (B), Tyr572 (B), Asp385 (A), Ser363 (B)
D-3	−7.3 kcal/mol	3	Tyr334 (B), Asn387 (A), Aeg483 (B)	Phe577 (B), Tyr572 (B), Ser383 (A), Asn382 (B), Ser383 (A), Ser363 (B), Ser555 (B), Arg414 (B), Arg380 (B), Ala556 (B), Arg415 (B), Gly462 (B), Ser508 (B), Tyr525 (B), Gln530 (B), Phe478 (B), His436 (B), Ile461 (B), Gly386 (A), Pro384 (A)
D-4	−9.0 kcal/mol	8	Pro384 (A), Ser383 (A), Asn387 (A), Arg415 (B), Tyr572 (B)	Gly509 (B), Ala556 (B), Gly603 (B), Asn382 (B), Tyr334 (B), Gly386 (A), Arg380 (A), Gln530 (B), Tyr525 (B), Arg483 (B), Ser508 (B), Phe478 (B), Gly462 (B)
D-5	−8.0 kcal/mol	7	Asn382 (B), Arg380 (B), Asn387 (B), Arg483 (B), Ser508 (B)	Gly364 (B), Asn414 (B), Phe577 (B), Tyr334 (B), Tyr572 (B), Gln530 (B), Gly509 (B), Tyr525 (B), Ser555 (B), Arg415 (B), Ser363 (B), Ala556 (B), Gly603 (B)
D-6	−7.4 kcal/mol	5	Ser383 (A), Ser602 (B), Arg415 (B), Asn414 (B), Pro384 (A)	Asp385 (A), Ser363 (B), Gly364 (B)Ala556 (B), Ser555 (B), Gly509 (B), Arg483 (B), Tyr525 (B), Ile461 (B), Phe478 (B)His436 (B), Gly433 (A), Arg380 (B), Gly386 (A), Tyr334 (B)

Note: (A) represents the amino acid on chain A of the receptor molecule, and (B) represents the amino acid on chain B of the receptor molecule.

**Table 3:** Peptide docking with ACE.

Number	Docking Energy	Number of Hydrogen Bonds	Hydrogen Bonds	Hydrophobic Interactions
D-2	−8.8 kcal/mol	16	Glu403, Ser516, Aeg522, Tyr360, Glu123, Arg124, Tyr62, Asn66, Asn70, Tyr523, Glu411, Zn701	Ser355, Phe391, His410, Pro407, Asp358, Trp59, Lys118, Ile88, Leu139, Val518, Glu143, Pro519, Trp357
D-4	−9.1 kcal/mol	7	Glu411, Arg522, Glu403, Zn701	His387, Glu384, Phe391, Val518, Asn356, Asn66, Trp357, Asp358, His410, Tyr360, Trp59, Tyr394

**Table 4:** Peptide docking with CE.

Number	Docking Energy	Number of Hydrogen Bonds	Hydrogen Bonds	Hydrophobic Interactions
D-2	−7.4 kal/mol	10	Phe60, His115, Arg63, Thr75, Gly106, Ser194, Gly107, Val108, Tyr123	Ile69, Met111, Glu193, His435, Ile439, Gln440, Ile323, Ala436, Asp437, Tyr125, Leu124, Phe119, Ala117, Asn118, Gly116, Lys62, Lys61
D-3	−6.9 kal/mol	6	Arg63, Asn121, Tyr125, Ile69, Ala108, Asn118	Phe119, Gly116, Gly112, Leu124, Met111, Gly107, Phe324, Ile323, Met281, Gly106, Glu193, Ile439, Ala117, Asp437, Ala436, Gln440
D-6	−7.0 kal/mol	8	Lys445, Tyr125, Ser194, His435, Gln440, Arg423	Asn121, Phe119, Asn118, Ala117, Gly116, Leu124, Arg63, Met111, Gly107, Ala108, Ile439, Glu193, Gly106, Ile323, Ala436

## Data Availability

The datasets generated during and/or analyzed during the current study are available from the corresponding author on reasonable request.

## Supplementary Materials

The following supporting information can be downloaded at: https://www.mdpi.com/article/10.3390/molecules28062589/s1, Figure S1: Mass spectrograms of 6 peptides; Figure S2: HPLC chromatograms of the 6 Synthetic peptides. (a): D-1; (b): D-2; (c): D-3; (d): D-4; (e): D-5; (f): D-6; Figure S3: Mass spectrum and structure of the 6 Synthetic peptides. (a): D-1; (b): D-2; (c): D-3; (d): D-4; (e): D-5; (f): D-6.

## Sample Availability

Samples of the compounds are available from the market.
