# Supplementary material for: Multiple Bioactivities of Peptides from Hydrolyzed Misgurnus anguillicaudatus
Source: Molecules. 2023 Mar 13;28(6):2589. doi: 10.3390/molecules28062589 (PMC10053552; doi:10.3390/molecules28062589)
Supplement: Supplementary file 1 [file molecules-28-02589-s001.zip › molecules-2256370-supplementary.pdf]

# Supplemental Information

# Multiple bioactivities of peptides from hydrolyzed *Misgurnus anguillicaudatus*

Baojie Dou[1,2] · Xudong Wu [1,2] · Zihan Xia [1,2] · Guanghao Wu [1,2] · Quanyou Guo[3] · Mingsheng Lyu [1,2*] and Shujun Wang [1,2,*]

1 Jiangsu Key Laboratory of Marine Bioresources and Environment/Jiangsu Key Laboratory of Marine Biotechnology, Jiangsu Ocean University, Lianyungang, 222005, China; e-mail@e-mail.com

2 Co-Innovation Center of Jiangsu Marine Bio-industry Technology, Jiangsu Ocean University, Lianyungang, 222005, China; e-mail@e-mail.com

2 East China Sea Fishery Research Institute, Chinese Academy of Fishery Sciences, Shanghai 200090, China; e-mail@e-mail.com

* Correspondence: mslyu@jou.edu.cn, sjwang@jou.edu.cn (M. Lyu and S. Wang）

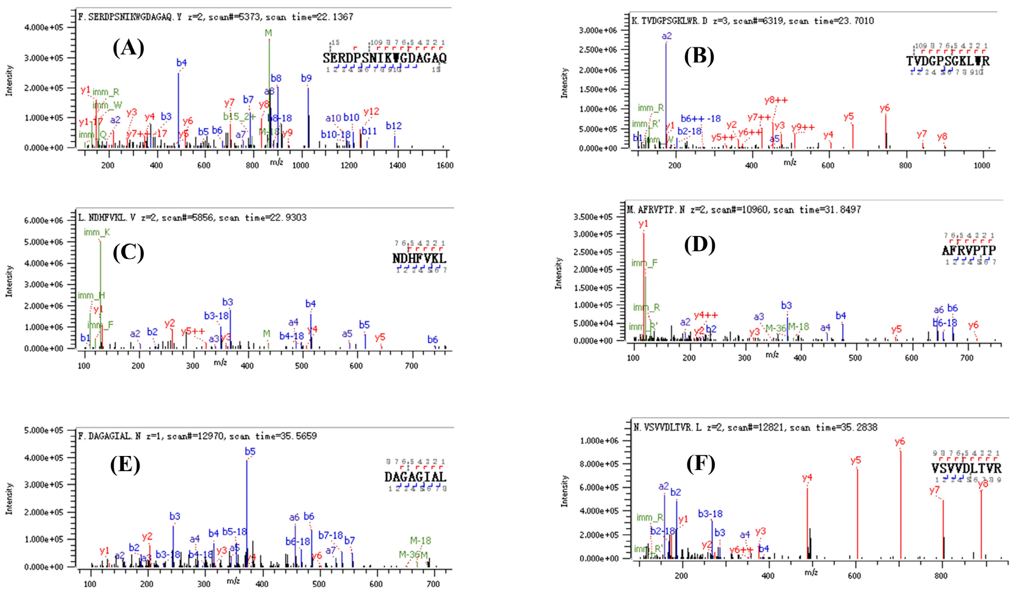


**Figure S1.** Mass spectrograms of 6 peptides.

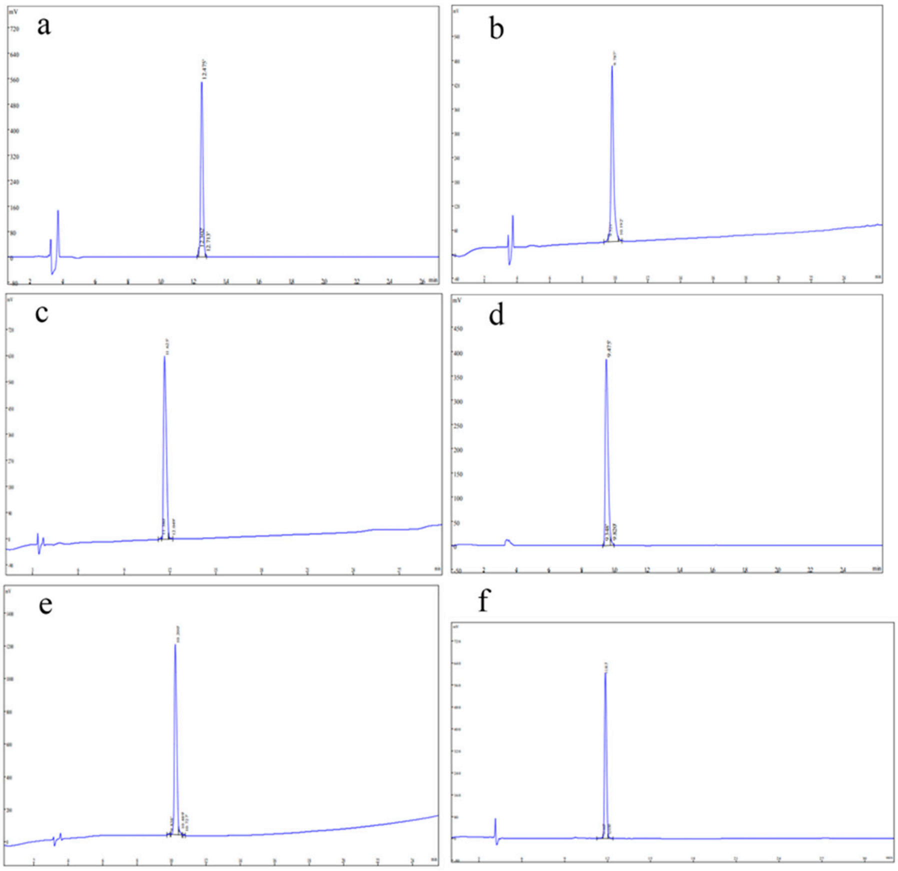


**Figure S2.** HPLC chromatograms of the 6 Synthetic peptides.

a:D-3;b:D-4;c:D-5;d:D-6;e:D-7;f:D-8

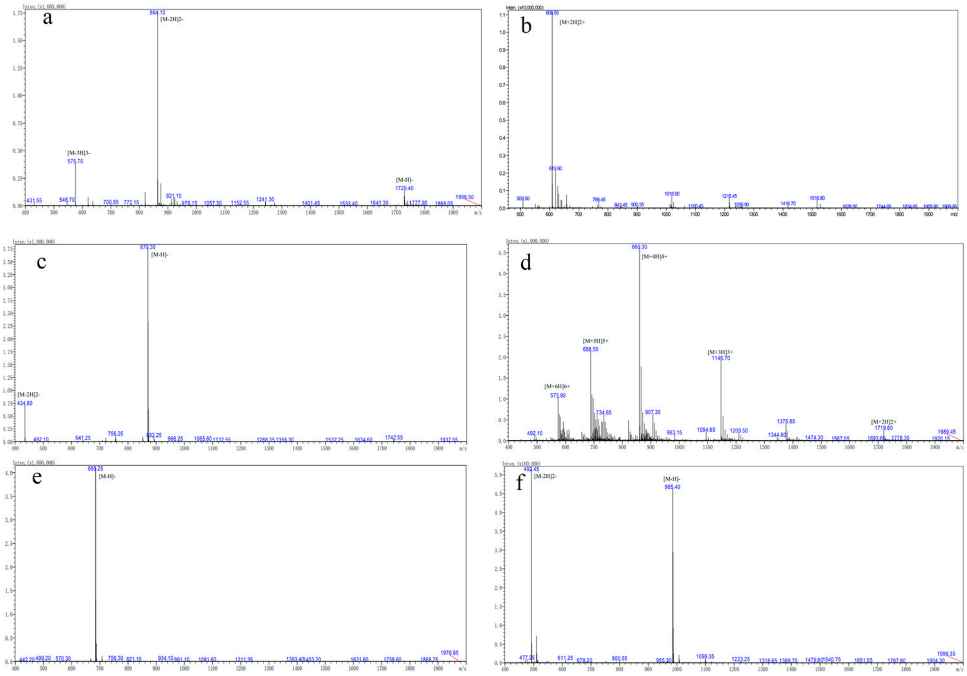


**Figure S3.** Mass spectrum and structure of the 6 Synthetic peptides.

a:D-3;b:D-4;c:D-5;d:D-6;e:D-7;f:D-8
